# Controllable two- and three-state magnetization switching in single-layer epitaxial Pd_1−_*_x_*Fe*_x_* films and an epitaxial Pd_0.92_Fe_0.08_/Ag/Pd_0.96_Fe_0.04_ heterostructure

**DOI:** 10.3762/bjnano.13.28

**Published:** 2022-03-30

**Authors:** Igor V Yanilkin, Amir I Gumarov, Gulnaz F Gizzatullina, Roman V Yusupov, Lenar R Tagirov

**Affiliations:** 1 Institute of Physics, Kazan Federal University, Kremlyovskaya Str. 18, 420008 Kazan, Russia; 2 Zavoisky Physical-Technical Institute, FRC Kazan Scientific Centre of RAS, 420029 Kazan, Russia

**Keywords:** anisotropic magnetoresistance, magnetization reversal, Pd–Fe alloy, thin epitaxial film

## Abstract

We have investigated the low-temperature magnetoresistive properties of a thin epitaxial Pd_0.92_Fe_0.08_ film at different directions of the current and the applied magnetic field. The obtained experimental results are well described within an assumption of a single-domain magnetic state of the film. In a wide range of the appled field directions, the magnetization reversal proceeds in two steps via the intermediate easy axis. An epitaxial heterostructure of two magnetically separated ferromagnetic layers, Pd_0.92_Fe_0.08_/Ag/Pd_0.96_Fe_0.04_, was synthesized and studied with dc magnetometry. Its magnetic configuration diagram has been constructed and the conditions have been determined for a controllable switching between stable parallel, orthogonal, and antiparallel arrangements of magnetic moments of the layers.

## Introduction

The generation of the long-range triplet component of the superconducting pairing at noncollinear orientations of magnetization in ferromagnetic layered systems is extensively studied in the framework of magnetic Josephson junctions (MJJs) ([1–3] for early works, and [4–5] for very recent papers), and superconductive spin valves (SSVs) [6–9]. The key points of the underlying physics are non-uniform magnetic configurations in the system that mix singlet and triplet superconducting pairing channels. As a result, at collinear magnetic configurations, short-range singlet and zero-spin-projection triplet pairings carry the Josephson supercurrent in MJJs. At non-collinear magnetic configurations, on the contrary, long-range equal-spin pairings can conduct the supercurrent in MJJs with much thicker or long narrow weak links. This gives additional degrees of freedom to control the critical current of MJJs [10] or SSVs [6], or current–phase relations in MJJs [11–12]. In particular, the spin-valve structure embedded into a MJJ can serve as an actuator for switching the MJJ between critical current modes or flipping its current–phase relation, thus, extending its functionality.

Palladium–iron alloys Pd_1−_*_x_*Fe*_x_* with *x* < 0.10 are of strong practical interest for such MJJ and SSV structures [13–17] as a material for weak ferromagnetic links with tunable magnetic properties [18]. Epitaxial films of Pd_1−_*_x_*Fe*_x_* alloys with low iron content *x* are easy-plane ferromagnets with four-fold anisotropy in the film plane [18–19]. Our conjecture is a possibility to switch the magnetic moment of a Pd_1−_*_x_*Fe*_x_* alloy film between the steady directions (90° apart) as it had been done with epitaxial iron films [20–22]. To realize this idea, it is necessary to find the particular angle of the applied magnetic field direction with respect to the in-plane four-fold easy axes. Once the conditions of magnetization rotation by 90° are found, the addition of the second, magnetically more hard ferromagnetic layer with properly aligned in-plane easy axes makes it possible to achieve parallel, orthogonal, and antiparallel configurations of their magnetic moments. Such a heterostructure can serve as a magnetic actuator for switching the MJJ from the singlet conduction mode to the triplet conduction mode and vice versa.

The experimental rotation of the magnetic moment by 90° in epitaxial Pd_1−_*_x_*Fe*_x_* films has not been yet explored. In [18–19], based on magnetometry data, it was assumed that magnetization reversal occurs as a result of the coherent rotation of the magnetic moment by 180°; and in the study of the Pd_0.96_Fe_0.04_/VN/Pd_0.92_Fe_0.08_ structure [23], stable parallel and antiparallel configurations of magnetic moments were obtained. However, the maximum amplitude of the triplet pairing component in the PdFe1/N/PdFe2 bilayer structure is achieved near the orthogonal magnetic configuration of the ferromagnetic layers PdFe1 and PdFe2 [6]. Therefore, it is instructive to investigate the switching properties of Pd_1−_*_x_*Fe*_x_* films and heterostructures based on this alloy targeting the controllable non-collinear magnetic configurations in the bilayer structure.

## Experimental

An epitaxial film of the Pd_0.92_Fe_0.08_ alloy with a thickness of 20 nm and an epitaxial thin-film heterostructure Pd_0.92_Fe_0.08_(20 nm)/Ag(20 nm)/Pd_0.96_Fe_0.04_(20 nm) were grown in an ultrahigh-vacuum (UHV) apparatus (SPECS, Germany) by molecular beam deposition. Epi-polished MgO(100) single-crystal plates (Crystal GmbH, Germany) were used as substrates. The deposition routine and structural studies of similar films are described in [24], the magnetic properties measured by ferromagnetic resonance (FMR) and vibrating sample magnetometry (VSM) in magnetic fields along the easy and hard magnetic axes are presented in [18–19].

In this paper, the magnetization reversal in the Pd_0.92_Fe_0.08_ film at different in-plane orientations of the magnetic field was studied by measuring the anisotropic magnetoresistance (AMR) using the four-probe method. For this purpose, the Pd_0.92_Fe_0.08_ film was cut with a diamond saw into stripe-like pieces. In the first sample the current flowed along the ⟨100⟩ direction of the Pd_0.92_Fe_0.08_ film (sample S00), in the second sample at an angle of 25° with respect to the ⟨100⟩ direction (sample S25). The current contacts were ultrasonically welded at several locations in a line across the width of the sample to ensure a uniform current distribution throughout the core part of the sample supplied with the potential terminals.

The magnetic hysteresis loops for the Pd_0.92_Fe_0.08_(20 nm)/Ag(20 nm)/Pd_0.96_Fe_0.04_(20 nm) heterostructure were obtained via vibrational sample magnetometry since the AMR measurement for this system is useless due to a short cut by the silver layer. All AMR and VSM experiments were carried out with the PPMS-9 system (Quantum Design).

## Results

### Сurrent along the [100] direction

**Figure 1:**
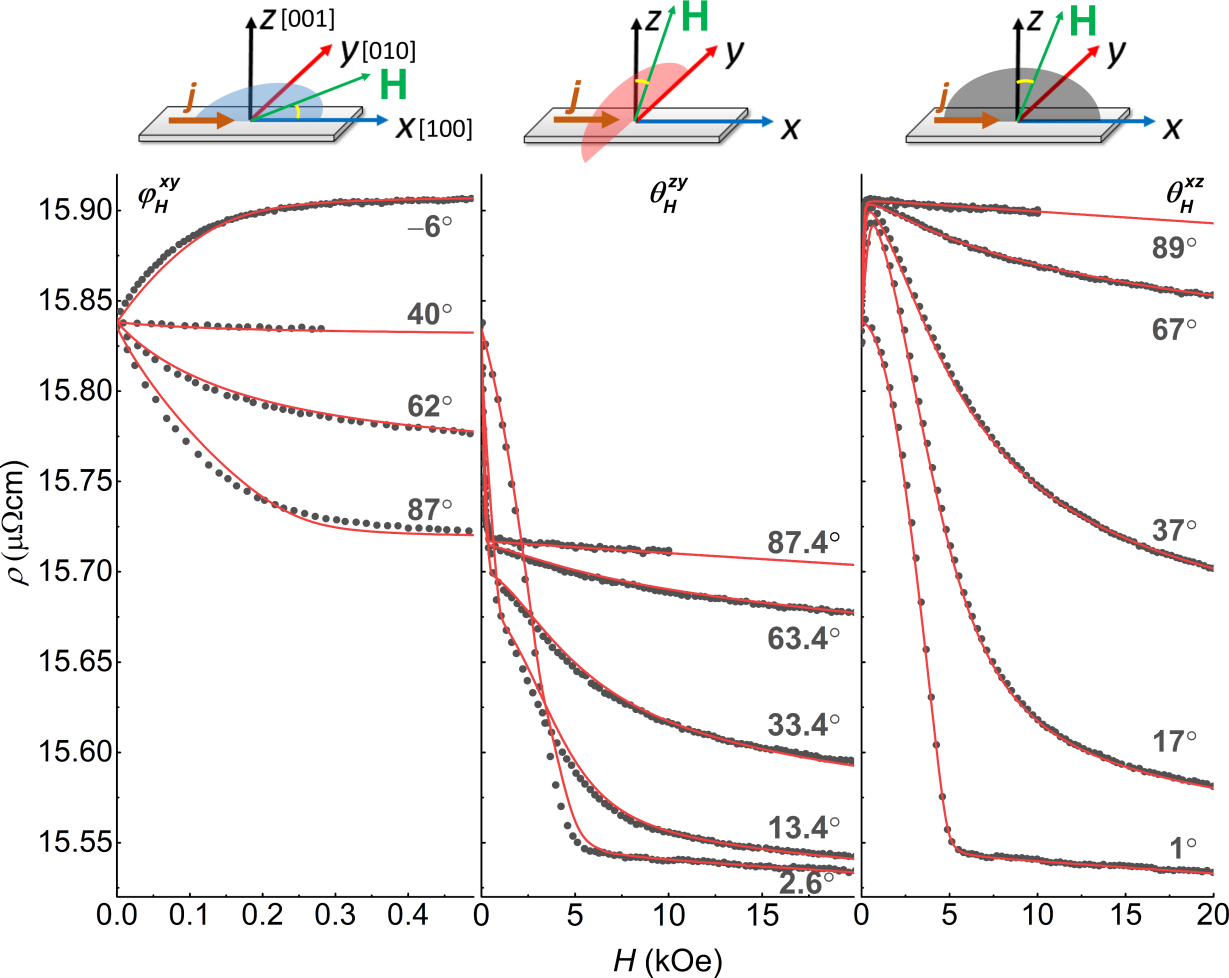
Dependence of the resistivity on the magnetic field at its different orientations, *T* = 5 K. The field is varied from its maximum value to zero. Circles indicate the experimental data, lines indicate the results of the modeling following Equation 1.

We start with the presentation of the results for a sample S00 of the Pd_0.92_Fe_0.08_ film where the electrical current flowed along the [100] direction of the film (and the substrate). The reference frame orientation with respect to the sample is shown in the insets in Figure 1. When measuring the magnetoresistance, the magnetic field was applied at different angles in three main planes as shown in Figure 1. At any orientation, on approaching zero field, the resistivity returns to a common value of approx. 15.8 μΩ·cm, corresponding to the magnetic moment along the easy axes (see more details below). The resistivities hierarchy for the magnetic moment oriented along the *X*-, *Y*-, and *Z*-axes, ρ*_x_* > ρ*_y_* > ρ*_z_*, is typical for ferromagnetic films of comparable thickness [25]. The difference in resistances ρ*_x_* and ρ*_z_* (magnetic field perpendicular to the current) is usually associated with the geometrical size effect [26]. A detailed study of the size effect for the Pd_0.92_Fe_0.08_ film is beyond the scope of this study.

**Figure 2:**
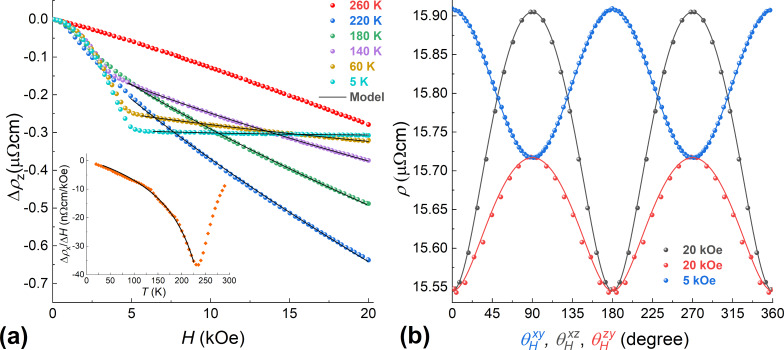
(a) Dependence of the resistivity Δρ*_z_* on the magnetic field strength at different temperatures. Inset: a variation of the (ρ*_x_*(10 kOe) − ρ*_x_*(0.5 kOe))/9.5 kOe slope with temperature. Lines are drawn using the model of Equation 2. (b) Dependence of the resistance on the orientation of the magnetic field at *T* = 5 K. The contribution from the electron–magnon scattering is subtracted. Lines are drawn using the model of Equation 1.

In addition to the anisotropic magnetoresistance associated with the mutual orientations of the magnetic moment and the direction of the current, there is a resistance drop with an increase of the magnetic field strength, that is, the negative magnetoresistance. The latter effect is related to a decrease in electron-magnon scattering due to the suppression of spin waves in high magnetic fields [27]. At low temperatures, this effect is usually small, and a positive magnetoresistance caused by the action of the Lorentz force dominates [27–28]. However, in the Pd_0.92_Fe_0.08_ epitaxial film, the electron mean free path is small even at low temperatures and is determined by the mean distance between the iron atoms. Therefore, the Lorentzian contribution is small, and even at 5 K, the negative magnetoresistance is observed. As the temperature is increased, the number of magnons grows, thereby leading to a larger negative slope Δρ/Δ*H* (Figure 2a). A theoretical description of this process for elemental 3d ferromagnets was proposed in [27]. The dependence of the resistance on a magnetic field up to 100 T is described by the expression:


[2] $$\Delta \rho_{m}=\rho_{m}\left(T,B\right)-\rho_{m}\left(T,0\right)\approx \frac{BT}{D\left(T\right)}\ln\left(\frac{\mu_{\text{B}}B}{kT}\right),$$

where *D*(*T*) ≈ *D*_0_ − *AT*^2^ determines the increase in the effective mass of magnons with increasing the temperature. The dependence 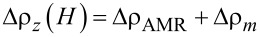 is shown in Figure 2a, where Δρ_AMR_ is the contribution from the anisotropic magnetoresistance. It is seen that Equation 2 describes the experimental results quite well. In the inset of Figure 2a, the variation of the slope of Δρ*_x_* with temperature is shown. It reaches the maximum value at the Curie temperature *Т*_С_ ≈ 230 K according to the magnetometry data.

At a fixed magnetic field and temperature, the resistance value reflects the direction of the magnetic moment in space. In a spherical coordinate system, the resistance is described by the following expression [29]:


[1] $$\begin{array}{c} \rho \left(\varphi_{M},\theta_{M},H\right)=\left(\rho_{x}\cos^{2}\varphi_{M}+\rho_{y}\sin^{2}\varphi_{M}\right)\sin^{2}\theta_{M}\\ +\rho_{z}\cos^{2}\theta_{M}+\Delta \rho_{m}\left(H\right), \end{array}$$

where ρ*_x_*, ρ*_y_*, and ρ*_z_* are the resistivities for the cases of the magnetic moment oriented along the *X*-, *Y*-*,* and *Z*-axes, respectively. The contribution from the electron–magnon scattering is subtracted for each of ρ*_x_*, ρ*_y_*, and ρ*_z_*. Angles θ*_M_* and φ*_M_* are referenced relative to the [001] and [100] axes, respectively.

**Figure 3:**
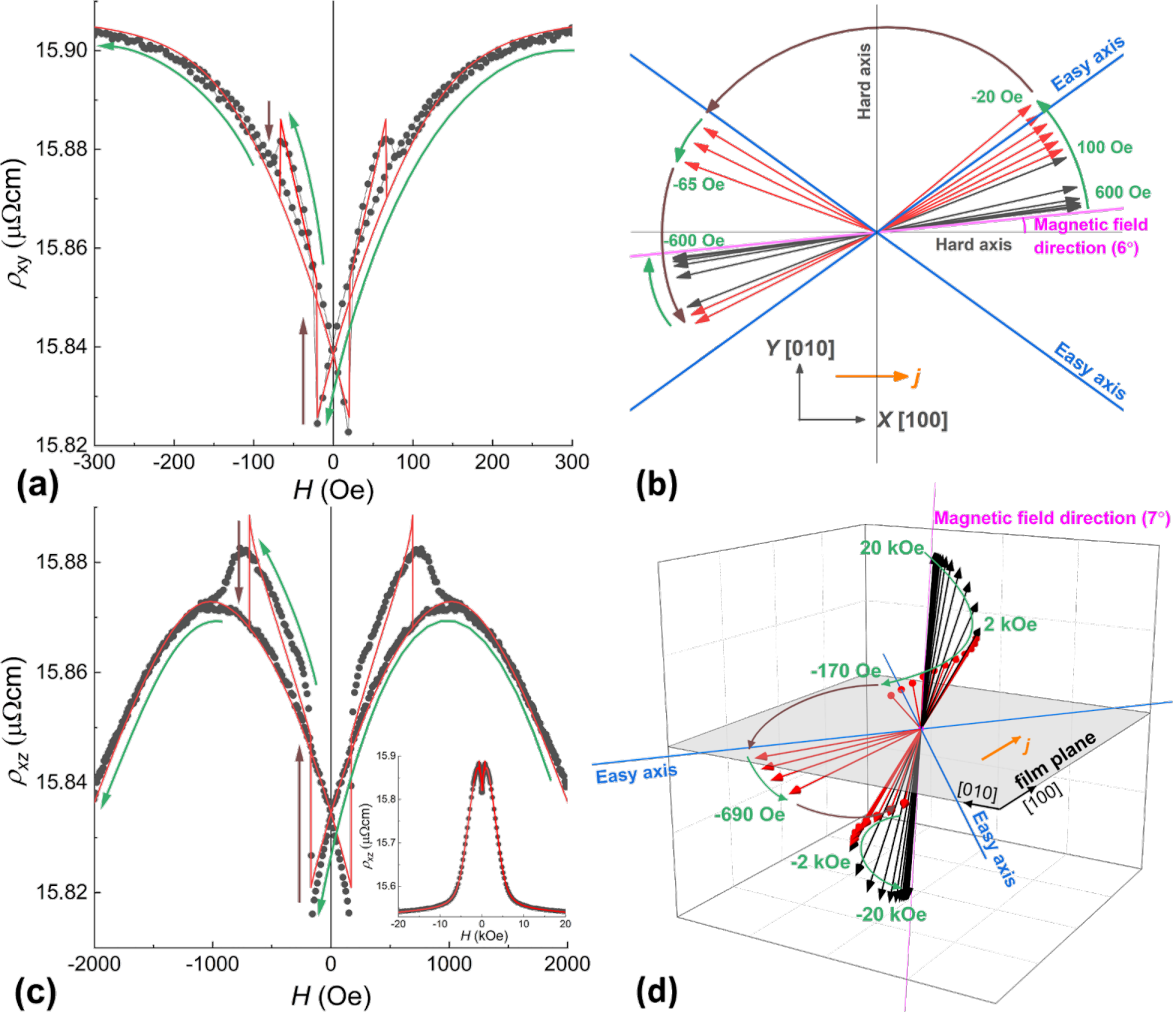
Dependences of the resistivity on the applied magnetic field for (a) φ*_Η_* = 6°, θ*_Η_* = 90° and (c) φ*_Η_* = 0°, θ*_Η_* = 7°; points: experimental data, lines: results of the modelling with Equation 1 and Equation 3. (b) Rotation of the magnetic moment of the film (within the same model) for φ*_Η_* = 6°, θ*_Η_* = 90°; the vectors show the direction of the moment at different values of the magnetic field (for the black vectors, the step is 100 Oe, while for the red ones it is −20 Oe); insert: the geometry of the experiment. (d) Rotation of the magnetic moment of the film (within the model) in space at φ*_Η_* = 0°, θ*_Η_* = 7°; (for the black vectors the step is 2 kOe, while for the red ones −0.2 kOe). Green arrows in all figures denote a smooth change in the direction of the magnetic moment, brown: magnetization direction jumps.

For a theoretical description of the dependencies ρ(*H*), ρ(φ*_H_*, θ*_H_*) presented in Figure 1 and Figure 2b, we used an approach similar to the Stoner–Wolfarth one for a single-domain magnetic system. According to [18], the Pd_0.92_Fe_0.08_ epitaxial film is the easy-plane system; the magnetocrystaline energy of the film can be written in terms of the cubic anisotropy with small tetragonal distortion. In addition, based on the guess simulations, an importance of the uniaxial anisotropy in the film plane became obvious:


[3] $$\begin{array}{c} E=-M\cdot H+2\pi M_{\text{s}}^{2}\alpha_{3}^{2}-K_{\text{p}}\alpha_{3}^{2}-K_{\text{u}}\alpha_{\text{u}}^{2}\\ -\frac{1}{2}K_{1}\left(\alpha_{1}^{4}+\alpha_{2}^{4}+\alpha_{3}^{4}\right)-\frac{1}{2}K_{2}\alpha_{3}^{4}\;, \end{array}$$

where α*_i_* are directional cosines for the magnetic *М* with respect to crystallographic axes [100], [010], [001] of the film, α_u_ is the cosine of the angle between *М* and the direction of the uniaxial anisotropy axis, *K*_u_ is the in-plane uniaxial anisotropy constant. As a result of the parameter adjustment, a good agreement of the theoretical dependences with the experimental data was achieved (Figure 1 and Figure 2b), indicating correctness of the models, Equation 1 and Equation 3. The values of the anisotropy constants, obtained by the fitting, are *K*_p_ = −190 kerg/cm^3^ for the perpendicular anisotropy term, *K*_1_ = −20 kerg/cm^3^ for the cubic term, and *K*_2_ = −20 kerg/cm^3^ for the tetragonal term, in good agreement with the values obtained from the FMR data analysis [18]. The uniaxial anisotropy is directed along [100], and its magnitude is *K*_u_ = 5 kerg/cm^3^. It should be noted that the occurrence of uniaxial anisotropy is not related to the shape of the samples. We further explored this by measuring the AMR and FMR of different square Pd_0.92_Fe_0.08_ samples and obtained the uniaxial anisotropy both along the [100] and [−110] axes. The origin of the uniaxial anisotropy term is not clear at the moment. Based on the values of the anisotropy constants, we can conclude that our film is a system with easy axes lying in the plane of the film and close to [110] (⟨110⟩) (approx. 38° relative to [100], see Figure 3b).

In the following, we denote the measured resistivity by ρ*_xy_*, ρ*_xz_*, and ρ*_yz_*, with the magnetic field applied in the *XY*-, *XZ*-, and *YZ*-planes, respectively. The angles are defined in the top insets in Figure 1. Let us consider in more detail the dependence of ρ*_xy_* on the magnetic field applied at φ*_H_* = 6° (Figure 3a). The measurement started from the saturation in the positive direction of the field (not shown). This direction is close to the hard axis; therefore, on the field value decrease, at its certain value of approx. 300 Oe, the magnetic moment starts to rotate coherently from the direction of the field towards the easy axis (see Figure 3a,b). At zero field, the moment is oriented along the easy axis. At small negative fields, the resistance experiences two abrupt jumps at −20 Oe and at −67 Oe. Such double jumps were observed in the ρ*_xy_*(*H*) dependence for a wide range of angles φ*_H_* of the applied magnetic field. The magnitude of the magnetic field, at which the jumps occur, depends on the direction of the field. Modeling of such a scenario (Figure 3a) brings us to the conclusion that these jumps in ρ*_xy_*(*H*) originate from two successive turns between the directions adjacent to the easy axes, as shown in Figure 3b.

The double jump also manifests itself in the dependences of ρ*_xz_*(*H*) and ρ*_yz_*(*H*). As an example, the dependence of ρ*_xz_* on the magnetic field applied at θ*_H_* = 7° and a modelled process of the magnetization reversal are shown in Figure 3c and 3d, respectively. Since the projection of the magnetic field onto the film plane is small (*H_x_* ≈ 0.12*H_xz_*), the coercive field values are increased and the double jumps becomes smeared.

### Current at an angle to [100] direction, field H in the *XY* plane

**Figure 4:**
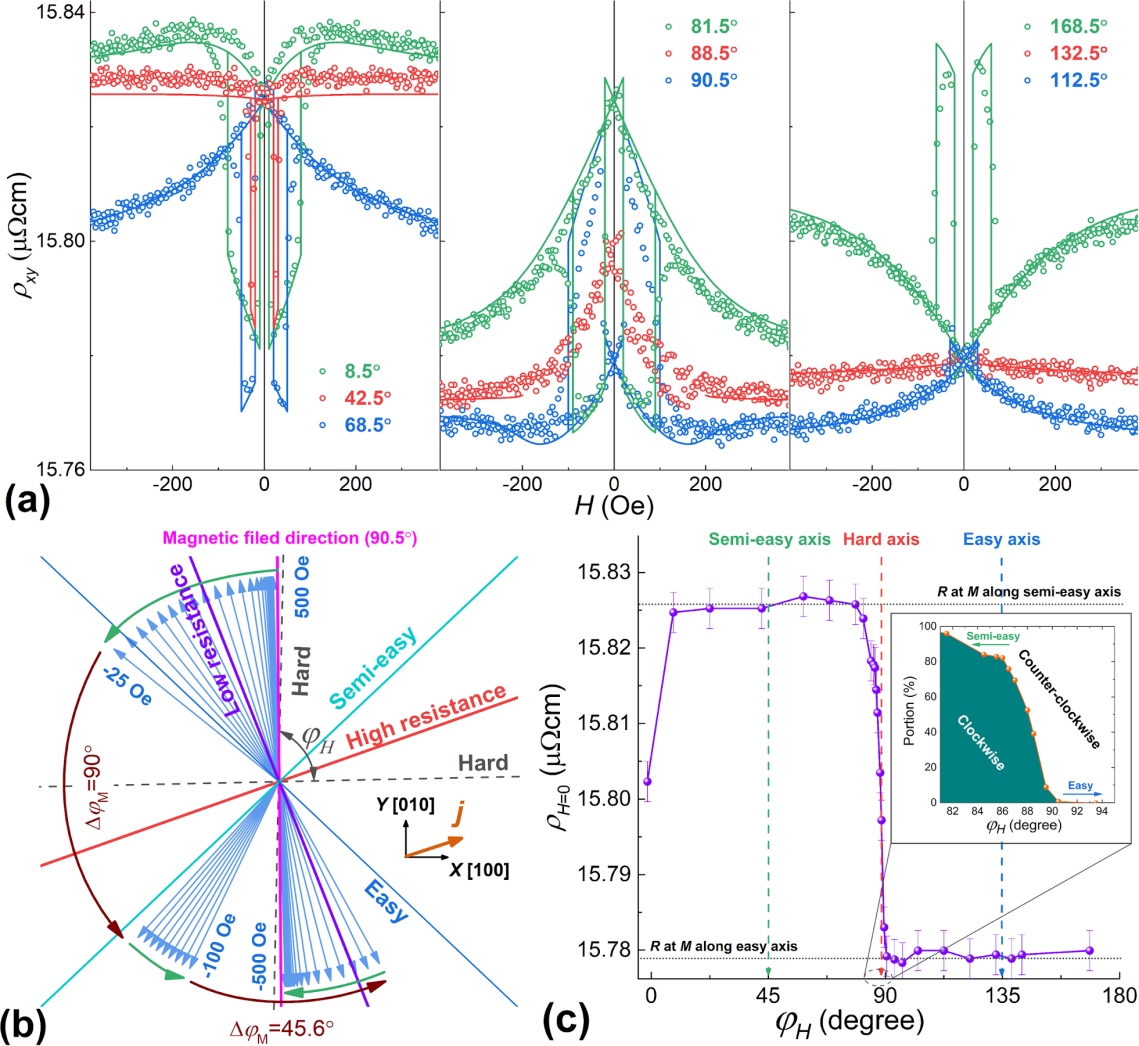
(a) Dependences of the resistivity ρ*_xy_* on the applied magnetic field for different angles φ*_H_* (measured from [100]) at *T* = 5 K; points: experimental data, lines: results of the modelling with Equation 1 and Equation 3. (b) Rotation (within the model) of the magnetic moment of the film with a step of 20 Oe for φ*_H_* = 90.5° under the field variation from +500 Oe to −500 Oe; insert: the geometry of the experiment. (c) Dependence of the resistance at zero fields on the direction of the applied field; insert: the calculated fraction of the film material with the magnetic moment rotated clockwise to a semi-easy axis.

For the sample S25, with a current directed at an angle of approx. 25° to the [100] axis, the double jumps are manifested much brighter (Figure 4a), since in this case, the easy axes are not equivalent in terms of the measured resistance. At the same time, the relative magnitude of the AMR effect (Δρ*_xy_*/ρ*_xy_*)·100% turns out to be less pronounced than with the current along the hard axis [100], that is, 0.45% and 1.22%, respectively. This is in a qualitative agreement with the experiments on AMR of epitaxial iron films, where the AMR effect was also maximal when the current direction was along the hard axis [30].

For the theoretical description of the ρ*_xy_*(*H*) dependences, the constant of the in-plane uniaxial anisotropy *K*_u_ and its direction were adjusted (leaving all the other parameters the same as for the previous sample). The easy axis of the uniaxial anisotropy was found along the [−110] direction (see the inset to Figure 4b) with *K*_u_ = 1 kerg/cm^3^. The model of the single-domain equilibrium state of the system describes well the experimental ρ*_xy_*(*H*) dependences (Figure 4a), except for the angles adjacent to the hard axis (see more details below).

A superimposed uniaxial anisotropy makes the [−110] direction easier than the [110] one. Therefore we call the latter the semi-easy axis. This significantly modifies the process of the magnetization reversal (compared with the first sample, where the easy axes were equivalent) when the magnetic field is applied along the easy axes. If the field is applied along the semi-easy axis, the magnetization reversal occurs in two jumps (Figure 4a, φ*_H_* = 42.5°), in the other case, the magnetization reversal occurs in one jump (Figure 4a, φ*_H_* = 132.5°). Recall that for AMR, the magnetic moment rotation by 180° does not lead to any change in resistance, therefore, a one-jump magnetization reversal manifests itself in an absence or only a small drop in resistance.

By measuring the residual resistance (after field removal), it is possible to realize which easy axis was chosen by a system (Figure 4c). Near the direction of the heavy axis, there is a transition from the counter-clockwise rotation to the clockwise one. This transition spans over a notable range of angles. For example, within the angle range of 82–90°, in the course of the magnetization reversal, one fraction of the film rotates its moment clockwise, while the other rotates counter-clockwise (Figure 4c, inset).

**Figure 5:**
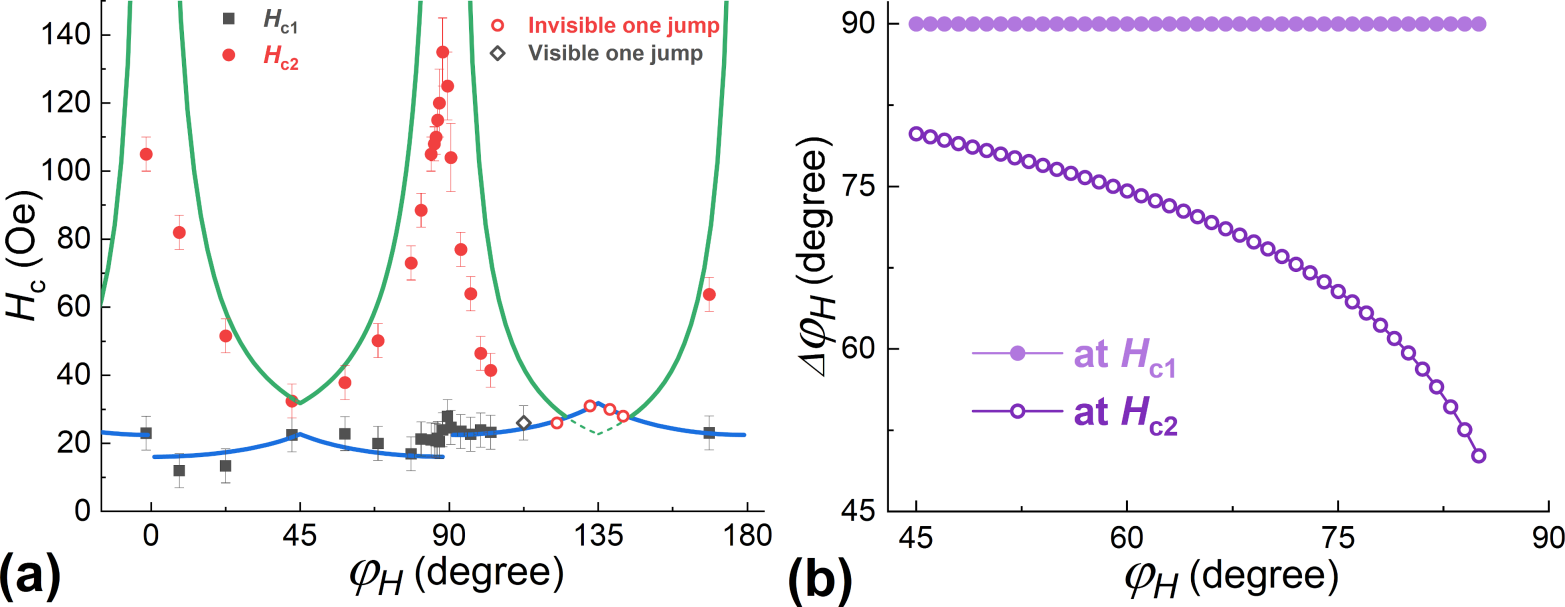
(a) Dependences of the coercive fields of the two jumps (*H*_c1_ and *H*_c2_) on the direction of the applied magnetic field at *T* = 5 K; lines: the model of Equation 4. (b) Dependence of the magnetic moment rotation angle within the particular jump on the direction of the applied field.

As follows from Figure 4a, the coercive fields corresponding to the first step of the reversal (*H*_c1_) and, especially, to the second one (*H*_c2_) depend significantly on the direction of the applied field. Figure 5a illustrates this relationship. A detailed explanation of this behavior was proposed in [21]. It suggested the successive movements of two 90° domain walls. The values of the coercive fields are determined by the conditions when the energy gain due to a moment rotation overcomes the wall pinning energy:


[4] $$H_{\text{c}1,\text{c}2}=\frac{\varepsilon_{90\deg}\pm K_{\text{u}}}{M\left(\pm \cos\left(\varphi_{H}-\frac{\pi }{4}\right)\pm \sin\left(\varphi_{H}-\frac{\pi }{4}\right)\right)}$$

For the Pd_0.92_Fe_0.08_ film, this model describes well the dependence of *H*_c1_(φ*_H_*) at ε_90deg_ ≈ 6 kerg/cm^3^. However, for the values of *H*_c2_, the 90° domain wall model is not suitable. This is because at the second jump the domain wall, in fact, is not the 90° type, the difference in the angles in the wall Δφ*_M_* is much smaller than 90° (see Figure 4b). Moreover, it depends on the angle of the applied field. Based on the experimental data for *H*_c2_(φ*_H_*), this dependence can be calculated. Figure 5b shows the obtained dependences Δφ*_M_*(φ*_H_*). It can be seen that as the hard axis is approached, the amplitude of the rotation angle of the moment in the domain wall decreases. In this situation, it is no longer possible to assert that the pinning energy is constant since it decreases with decreasing the angle difference in the domain wall.

For the field directions close to the hard axis (for example, at φ*_H_* = 87–90°), it is generally difficult to describe the magnetization reversal, since the volume of the material is divided into two fractions that rotate their moments in opposite directions. In addition, at the field strengths of the second jump in magnetization, the height of the barrier for the coherent rotation also becomes insignificant. Therefore, in this range of angles, magnetization reversal is potentially possible by the macroscopic coherent rotation of the magnetic moment of the film.

## Discussion

**Figure 6:**
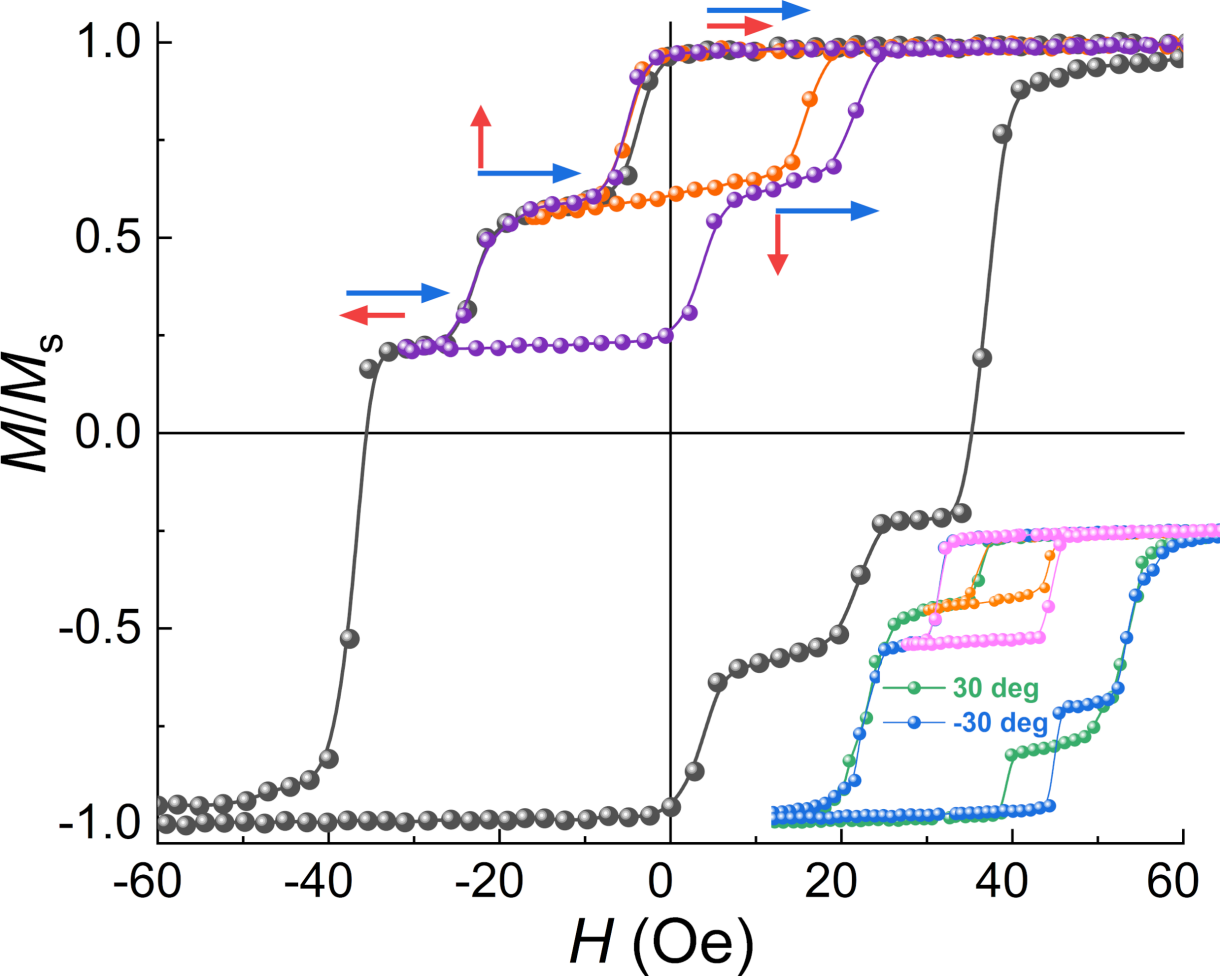
Magnetic hysteresis loops for the Pd_0.92_Fe_0.08_/Ag/Pd_0.96_Fe_0.04_ heterostructure at *T* = 5 K at an angle of 40° with respect to [100] (inset: the same for angles of 30° and −30°).

**Figure 7:**
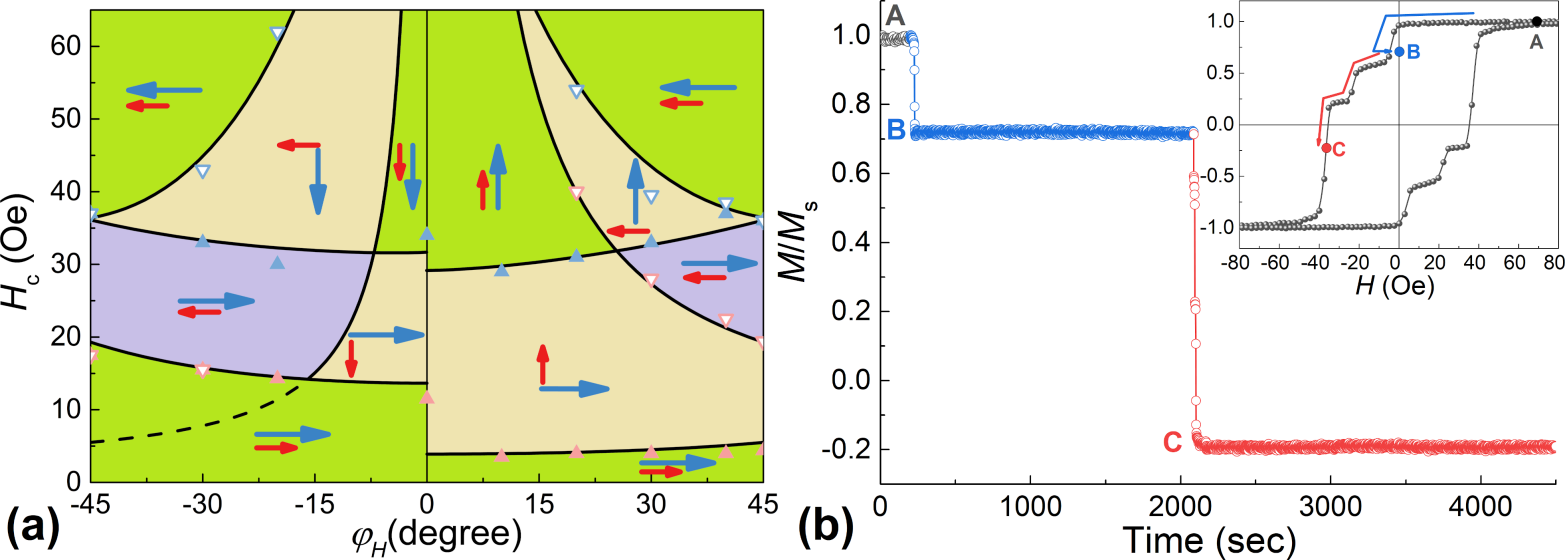
(a) Dependences of the coercive fields of the Pd_0.92_Fe_0.08_/Ag/Pd_0.96_Fe_0.04_ heterostructure on the angle of the applied magnetic field relative to the [100] axis at *T* = 5 K. The symbols are experimental values (red: Pd_0.96_Fe_0.04_, blue: Pd_0.92_Fe_0.08_, solid: *H*_c1_, open: *H*_c2_), the lines represent the calculations. (b) Time dependence of the reduced moment for a magnetic field at an angle of 40° to [100]; arrows in the inset show the path from point A (P) to points B (OG) further to C (mixed AP+OG).

The dependences of *H*_c1,c2_(φ*_H_*) obtained in this work for the Pd_0.92_Fe_0.08_ film confirm the conjecture that three steady magnetic configurations, parallel (P), anti-parallel (AP), and orthogonal (OG) can be realized in the epitaxial PdFe1/N/PdFe2 heterostructure by choosing the appropriate magnetic field direction and varying the applied magnetic field pulse amplitude. An important condition for this is an absence of a substantial magnetic interaction between the PdFe1 and PdFe2 layers. It is achieved by introducing the non-magnetic spacer layer N of silver satisfying the epitaxial growth conditions. The different coercive fields were obtained choosing the Pd_0.92_Fe_0.08_ and Pd_0.96_Fe_0.04_ compositions for the ferromagnetic PdFe1 and PdFe2 layers [18]. Figure 6 shows magnetic hysteresis loops of the Pd_0.92_Fe_0.08_/Ag/Pd_0.96_Fe_0.04_ heterostructure for magnetic field angles of 40°, 30°, and −30° degrees relative to the [100] direction. For an angle of 40°, the minor loops show the switching sequence of P → OG → AP, for 30°, P → OG, and for −30°, P → AP. The analysis of the magnetic hysteresis loops at different angles of the applied magnetic field makes it possible to build a magnetic configuration diagram for this heterostructure (Figure 7a). As one can see, there are quite large regions of various stable P, OG, and AP states.

An analysis of the angular dependences of *H*_c_(φ*_H_*), Figure 7a, allows us to conclude that the pinning energy for the Pd_0.94_Fe_0.04_ film is much lower (about ε_90deg_ ≈ 1.8 kerg/cm^3^) than for the Pd_0.92_Fe_0.08_ film (ε_90deg_ ≈ 6 kerg/cm^3^). Moreover, in our Pd_0.92_Fe_0.08_/Ag/Pd_0.96_Fe_0.04_ heterostructure, the Pd_0.96_Fe_0.04_ layer has a significant uniaxial anisotropy (*K*_u_ ≈ 1 kerg/cm^3^) along the [−110] direction, while in the Pd_0.92_Fe_0.08_ layer it is practically absent.

Low temperatures and single-domain state (or bi-domain at magnetization reversal along a hard axis) of the magnetic film ensure the stability of the obtained magnetic configurations. Figure 7b shows the time evolution of the reduced moment depending on the history of the evolution of the applied magnetic field (inset to Figure 7b). The system was brought to point B (OG state) by sweeping the magnetic field in the following order: 50 Oe → −5 Oe → 0 Oe; to point C (mixed AP+OG state): 0 Oe → −36 Oe. In both cases, on a time scale of approx. 1000 s, there are no noticeable changes in the magnetic moment of the steady states of the heterostructure. This is fundamentally different from the dynamics of the magnetization of the polycrystalline Pd_0.99_Fe_0.01_ film, in which significant demagnetization occurred on a time scale of approx. 100 s [31].

## Conclusion

Detailed measurements of the magnetoresistance have shown that the Pd_0.92_Fe_0.08_ epitaxial film, being an easy-plane ferromagnet with a pronounced in-plane anisotropy, undergoes magnetization switching between two (with collinear magnetization directions) or three (including orthogonal to the previously indicated two directions) single-domain states depending on the direction of the applied magnetic field. In the latter case, the magnetization reversal proceeds in two distinct stages, the first stage being the motion of the 90° domain wall, and the second one is the motion of the angle-φ domain wall, where the angle φ depends on the angle of the applied field relative to crystallographic axes. The pinning energy of the 90° domain wall is approx. 6 kerg/cm^3^ for the Pd_0.92_Fe_0.08_ film, and approx. 1.8 kerg/cm^3^ for the Pd_0.96_Fe_0.04_ film. The use of two magnetic layers PdFe1 and PdFe2 with different coercive fields, separated by a nonmagnetic spacer N, makes it possible to realize parallel, orthogonal, and antiparallel configurations of magnetic moments. It has been experimentally demonstrated that the Pd_0.92_Fe_0.08_/Ag/Pd_0.96_Fe_0.04_ heterostructure can switch between P, OG, and AP steady magnetic configurations in the film plane by rotating the magnetic moment of the soft magnetic layer with respect to the magnetically harder layer.
